# Detection of KPC-producing *Enterobacterales* species in wastewater samples from the Gran Concepción Metropolitan area, Chile

**DOI:** 10.1186/s40659-025-00612-7

**Published:** 2025-06-07

**Authors:** Franco Ilabaca-Carrasco, Carlos Peña-Raddatz, Claudia Torres-Bustos, Mauricio Hernández-Cea, Guillermo Nourdin-Galindo, Pablo Saldivia-Flandez, Cristian Vargas, Elard Koch, Helia Bello-Toledo, Gerardo González-Rocha, Andrés Opazo-Capurro

**Affiliations:** 1 Laboratorio de Investigación en Agentes Antibacterianos (LIAA), Departamento de Microbiología, Facultad de Ciencias Biológicas, Universidad de Concepción, Concepción, Chile; 2 Grupo de Estudio en Resistencia Antimicrobiana (GRAM), Universidad de Concepción, Concepción, Chile; 3 Laboratorio de Biología, Departamento de Ciencias Biológicas, Facultad de Ciencias de La Vida, Universidad Andrés Bello Sede Concepción, Talcahuano, Chile; 4 Division of Biotechnology, MELISA Institute, Concepción, Chile

**Keywords:** Antibiotic resistance, Wastewater, Carbapenem-resistant *Enterobacterales*, KPC-type carbapenemases, One health

## Abstract

**Background:**

Carbapenemase-mediated resistance to carbapenems is a significant public health concern due to its potential for widespread dissemination. The KPC family of carbapenemases, encoded by the *bla*_KPC_ gene and often associated with Tn*4401*-like transposons, is particularly important for its ability to be transferred through diverse plasmid types. In Chile, KPC-producing Gram-negative bacteria have been detected in clinical settings; however, their occurrence in wastewater (WW) remains unknown. This study addresses this gap by characterizing carbapenem-resistant *Enterobacterales* isolates from a wastewater treatment plant (WWTP) in the Gran Concepción Metropolitan Area, Chile.

**Results:**

This study identifies three carbapenem-resistant *Enterobacterales* isolates, namely *Klebsiella pasteurii* M2/A/C/34, *Klebsiella pneumoniae* subsp. *pneumoniae* M3/A/M/3, and *Citrobacter freundii* sensu stricto. M4/A/C/32, all exhibiting multidrug-resistant profiles and carrying the *bla*_KPC-2_ gene encoding KPC-like carbapenemases. These isolates also possessed genes for extended-spectrum β-lactamases (ESBLs) and aminoglycoside-modifying enzymes (AMEs). Sequence typing revealed that M2/A/C/34, M3/A/M/3, and M4/A/C/32 belonged to novel sequence types, specifically ST470, ST273, and ST214, respectively. All isolates carried plasmids belonging to groups commonly associated with ARGs, including IncF, IncP, and IncA. Both *Klebsiella* isolates (M2/A/C/34 and M3/A/M/3) carried the class 1 integron (*intl1*) gene. Phylogenomic analysis reveals that M2/A/C/34 is related to strains from China and Pakistan, while M3/A/M/3 shares similarities with a strain from Germany, indicating their potential dissemination.

**Conclusions:**

This study represents the first detection of carbapenem-resistant *Enterobacterales* carrying *bla*_KPC-2_ in Chilean WW, including the novel identification of *K. pasteurii*. These findings emphasize the critical role of genomic surveillance in WW under the One Health framework, enabling the monitoring of carbapenemase-producing bacteria and associated ARGs. Sustained surveillance efforts are essential to comprehend the dynamics of antibiotic resistance in environmental reservoirs and to develop strategies for its containment and mitigation.

**Supplementary Information:**

The online version contains supplementary material available at 10.1186/s40659-025-00612-7.

## Background

Antimicrobial resistance (AMR) poses a significant health challenge across human, animal, and environmental interfaces, demanding comprehensive investigation through the One Health approach. Recent studies estimate that antibiotic-resistant bacteria (ARB) caused 1.3 million deaths worldwide in 2019 [1]. Without effective measures, AMR is projected to result in up to 10 million deaths annually by 2050, accompanied by substantial economic consequences, underscoring the urgent need for global action to mitigate its impact [1], highlighting its profound impact on public health.

The World Health Organization (WHO) recently published a list of antibiotic-resistant pathogens, highlighting carbapenem-resistant Gram-negative bacteria as a ‘critical priority’. This designation emphasizes the urgent need for research and development of new antimicrobial therapies to address these highly resistant and clinically significant pathogens [2, 3]. Carbapenemase-mediated resistance is regarded as the most critical mechanism of carbapenem resistance, largely because of its capacity for rapid dissemination through mobile genetic elements (MGEs), such as plasmids and transposons. This mobility facilitates the widespread transmission of resistance genes across diverse bacterial species and environments, posing a significant threat to global public health [4]. Among these enzymes, *Klebsiella pneumoniae* carbapenemases (KPC) represent one of the most prevalent carbapenemases groups worldwide, initially associated to the clonal expansion of the *K. pneumoniae* ‘high-risk’ clone ST258 [5]. Furthermore, the horizontal transmission of KPC enzymes has been documented, with the *bla*_KPC_ gene typically associated with different isoforms of the transposon Tn*4401*, notably Tn*4401a* and Tn*4401b* [5]. These transposons are often embedded into various plasmid types, predominantly IncFII, IncL/M, and IncN [5], facilitating the horizontal spread of *bla*_KPC_.

The environmental dissemination of carbapenem-resistant bacteria represents a major public health concern and a critical challenge within the One Health framework [6]. ARB originating from urban environments eventually reach wastewater treatment plants (WWTPs), which can serve as reservoirs for both ARB and antibiotic resistance genes (ARGs) [7]. Wastewater (WW) surveillance has been proposed as an effective strategy for monitoring AMR at the population level within the One Health framework. This approach provides a comprehensive reflection of the epidemiological trends of AMR within specific populations, such as cities, while circumventing ethical concerns associated with individual sampling [8]. In this context, KPC-producing Gram-negative bacteria have been detected in WW samples, known to be significant reservoirs of ARGs and ARB [9–11]. In Chile, the first description of KPC was reported in 2012, in a *K. pneumoniae* strain recovered from a patient’s rectal swab who had traveled to Italy [12]. Since then, several reports of KPC-producing bacteria have been documented [13–18], however, these reports are primarily focused on clinical isolates.

Despite the identification of KPC-producing bacteria in Chile, there are no reports of these bacteria in WW samples. Therefore, this study aimed to characterize three carbapenem-resistant *Enterobacterales* isolates collected from a WWTP in the Gran Concepción Metropolitan area. This WWTP collects WW from the third most populated region in Chile with 958.034 inhabitants (https://www.subdere.gov.cl/sala-de-prensa/dos-nuevas-%C3%A1reas-metropolitanas-se-constituyen-oficialmente-santiago-y-el-gran), which constitutes ca. 19.3% of the total population of Chile that encompasses households, nursing homes, hospitals, industries in Gran Concepción Metropolitan Area.

## Methods

### Sample collection and storage

Eight WW samples were collected from a WWTP situated in the Gran Concepción Metropolitan area, encompassing ten cities in the Biobio Region, Chile. Sampling occurred in January, April, and July of 2022, with each sample comprising 1 L of wastewater collected over a 24 h period from both influent (IF) and effluent (EF) streams (four IF and four EF samples). All samples were preserved at a temperature of 2–8 °C and processed the day after receipt to guarantee sample integrity.

### Samples processing

EF samples of 1L were filtered through 0.45 µm Whatman^®^ filters with an Oil-Free Vacuum Pump Rocker 300 system (Rocker Scientific, USA). A secondary filtration step was performed using 0.22 µm Whatman^®^ filters. The 0.22 µm filters were then transferred into individual 50 mL Falcon tubes, where 5 mL of sterile distilled water was added. The filters were vortexed for 10 min to release retained materials and stored at 4 °C for subsequent analysis. In contrast, IF samples were processed directly from the sampling bottles without prior filtration.

### Bacterial isolation

IF samples were serially diluted directly in sterile water [19]. Aliquots of 0.1 mL from both undiluted samples and each dilution were subsequently inoculated onto MacConkey agar plates (MCC) and MacConkey agar plates supplemented with 2 µg/mL meropenem (MCC + M) to assess the presence of carbapenem-resistant isolates. Additionally, the filtered samples of EF were resuspended in sterile water and processed similarly to IF samples. All plates were incubated overnight at 35 °C. After incubation, the growth on both MCC and MCC + M was examined. Three to five morphologically similar colonies were then selected for further analysis. These colonies were sub-cultured on tryptone soy agar (TSA) and subsequently transferred to tryptone soy broth (TSB). The samples were then incubated overnight at 35 °C before cryopreservation at − 80 °C in a 2:1 solution of broth and glycerol (50% v/v) [20].

### Antibiotics susceptibility testing and determination of the minimum inhibitory concentration (MIC)

Antibiotic susceptibility testing of the colonies grown on MCC + M was performed using the agar diffusion method, in accordance with the guidelines of the Clinical and Laboratory Standards Institute (CLSI) [21]. The antibiotics tested included cefoxitin (FOX) (30 µg), ceftriaxone (CRO) (30 µg), ceftazidime (CAZ) (30 µg), cefotaxime (CTX) (30 µg), cefepime (FEP) (30 µg), imipenem (IPM) (10 µg), ertapenem (ETP) (10 µg), meropenem (MEM) (10 µg), gentamicin (GEN) (10 µg), amikacin (AMK) (30 µg), ciprofloxacin (CIP) (5 µg), levofloxacin (LVX) (5 µg), chloramphenicol (CHL) (30 µg), tetracycline (TET) (30 µg), sulfonamides (SUL) (300 µg), and trimethoprim (TRI) (5 µg). *Escherichia coli* ATCC 25922 was used for quality control. The minimum inhibitory concentration (MIC) of IMP for carbapenemase-producing isolates was determined using epsilomectric strips (Liofilchem®, Italy), following the manufacturer’s instructions.

From the susceptibility results, we calculated the multiple antibiotic resistance index (MAR), which is calculated as MAR index = a/b, where a = number of isolates resistant to antibiotics and b = total number of antibiotics tested [22, 23]. Antibiotic resistance profiles were classified according to Pearson et al*.* [24], which categorizes bacterial isolates as multidrug-resistant (MDR), extensively drug-resistant (XDR), or pandrug-resistant (PDR) based on their resistance to different antibiotic groups. According to this classification, MDR *Enterobacterales* are resistant to at least 3 out of 12 antibiotic groups, while XDR isolates exhibit resistance to 10 or 11 of the 12 groups, and PDR isolates are resistant to all 12 groups.

### Phenotypic detection of carbapenemases

Carbapenemase production was assessed using the BlueCarba test [25] on isolates (N = 3) that grew on MCC + M plates. To confirm the BlueCarba test results, the isolates were further evaluated using the NG-Test^®^ CARBA-5 immunoassay (NG Biotech, France), following the manufacturer’s protocol.

### Whole-genome sequencing (WGS) of carbapenemase-producing *Enterobacterales* strains and in silico analyses

Genomic DNA (gDNA) from the three carbapenemase-producing isolates was extracted using the DNeasy UltraClean Microbial kit (Qiagen). The purity of gDNA samples was assessed with NanoQuant spectrophotometry, and concentration was determined using the Qubit^®^ 3.0 dsDNA BR assay. Genomic libraries were prepared from 400 ng of gDNA using the Illumina DNA Prep Kit (Illumina) and Nextera DNA CD indexes (Illumina), following the manufacturer’s protocol. Library quantification was performed with Qubit 3.0, and library integrity was verified by capillary electrophoresis using the Qsep-100 (Bioptic Inc.). The libraries were then subjected to whole genome sequencing (WGS) on the NextSeq 500 sequencing platform (Illumina) with the NextSeq 500/550 High Output Kit v2.5 of 150 cycles (paired-end, 2 × 75 bp read length). Following sequencing, reads were demultiplexed using the BaseSpace application (Illumina). Quality assessment of the reads was conducted using FastQC software (https://www.bioinformatics.babraham.ac.uk/projects/fastqc/), and low-quality reads and adapters were filtered using Trimmomatic v0.36 [26] with IlluminaClip step. For genome analyses, filtered raw reads were assembled into contigs using Unicycler v0.5.1 [27], and sequencing statistics were obtained utilizing Quast v5.2.0 [28] and BUSCO v5.8.0 [29, 30] (Table S1). Gene annotations were performed through Bakta v1.9.4 [31].

Species identification of the three carbapenemase-producing isolates was conducted using the Ribosomal Multilocus Sequence Typing (rMLST) tool [32] and the JSpeciesWS [33] with tetracorrelation search (TCS). Subsequently, reference genomes corresponding to the bacterial species with the highest z-scores were retrieved from GenBank [34], and their average nucleotide identity percentages were determined using the BLAST + algorithm (ANIb%). Finally, each group of genomes was analyzed using the Genome-to-Genome Distance Calculator 3.0 (GGDC) platform (Leibniz Institute DSMZ) [33, 34] to calculate in silico DNA-DNA hybridization percentages (DDH%). The reference genomes used are listed in Table S1.

ARGs were identified using ABRIcate v1.0.1 [37] available on the Galaxy Australia platform (https://usegalaxy.org.au/) utilizing ResFinder v4.4.2 [38] and the NCBI Bacterial Antimicrobial Resistance Reference Gene Database. The sequence types (STs) of the isolates were determined using the MLST v2.0.9 tool [39] and mobile genetic elements (MGEs) associated with ARGs were identified using the Mobile Element Finder v1.0.3 tool [40], whereas integrons were detected utilizing IntegronFinder v2.0.3 [41]. Additionally, plasmids were re-constructed using the MOB Suite tools v3.1.9 [42], and Inc groups were determined by PlasmidFinder v2.1 [43] (Table 3).

For the two out of the genomes belonging to the *Klebsiella* genus, further analysis was conducted using cgMLST [44], Kleborate v2.3.2 [45] and Kaptive v2.0.7 [46] tools available on the PathogenWatch server (https://pathogen.watch/). The remaining genome (1/3) belonging to the *Citrobacter* genus, underwent analysis using the virulence factor database (VFDB) [47] via ABRIcate v1.0.1.

### Genomic epidemiology of the carbapenemases-producing strains

For epidemiological analyses, we retrieved genomes from Chilean strains of the genera *Klebsiella* with the same STs from the Bacterial and Viral Bioinformatics Resource Center (BV-BRC) [48] and PathogenWatch databases [46, 47]. Due to the absence of genomes of *Citrobacter* of the same ST, we determined its taxonomic classification in the Genome Taxonomy Database (GTDB) by the GTDB-Tk v2.5.1 tool [51] available on Galaxy Australia website.

Genomes were annotated using Bakta v1.5.0 [31], followed by alignment of core genomes using Roary v3.13.0 [52] and filtering by SNP-sites [53]. The phylogenomic trees were constructed using the maximum likelihood (ML) method with IQ-TREE v2.1.2 [54], and the resulting trees were visualized and edited using IToL v6.8.2. [55]. Additionally, we generate two SNP matrix from the output file generated by SNP-sites v2.5.1 [53], by using SNP-distance Matrix v0.8.2 tool (https://github.com/tseemann/snp-dists), available on the Galaxy Australia website. Finally, the matrix values were plotted in Heatmap format using Seaborn and Matlab. (Figures S1 and S2).

## Results

**Fig. 1:**
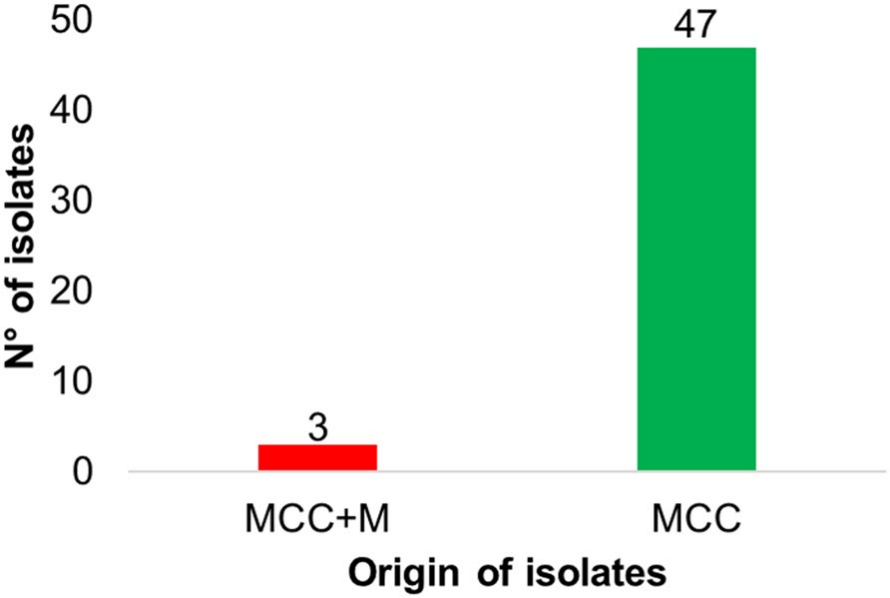
Distribution of oxidase-negative glucose-fermenting Gram-negative bacilli (Ox[–]-F-GNB) obtained from wastewater samples (WW). MCC: MacConkey agar, MCC + M: MacConkey agar supplemented with meropenem (2 µ/mL)

A total of 205 Gram-negative isolates were obtained from the WW samples analyzed. Among these, 50 were identified as oxidase-negative, glucose-fermenting Gram-negative bacilli (Ox[-]-F-GNB) based on their phenotypic characteristics observed on MCC plates and confirmed by Gram staining. Of these 50 isolates, 48 were recovered from IF, while 2 were isolated from EF. Additionally, 3 isolates from IF were identified on MCC + M plates, while no isolates from EF were recovered on MCC + M plates (Fig. 1). These 3 isolates from MCC + M, were included in subsequent analyses.

**Fig. 2:**
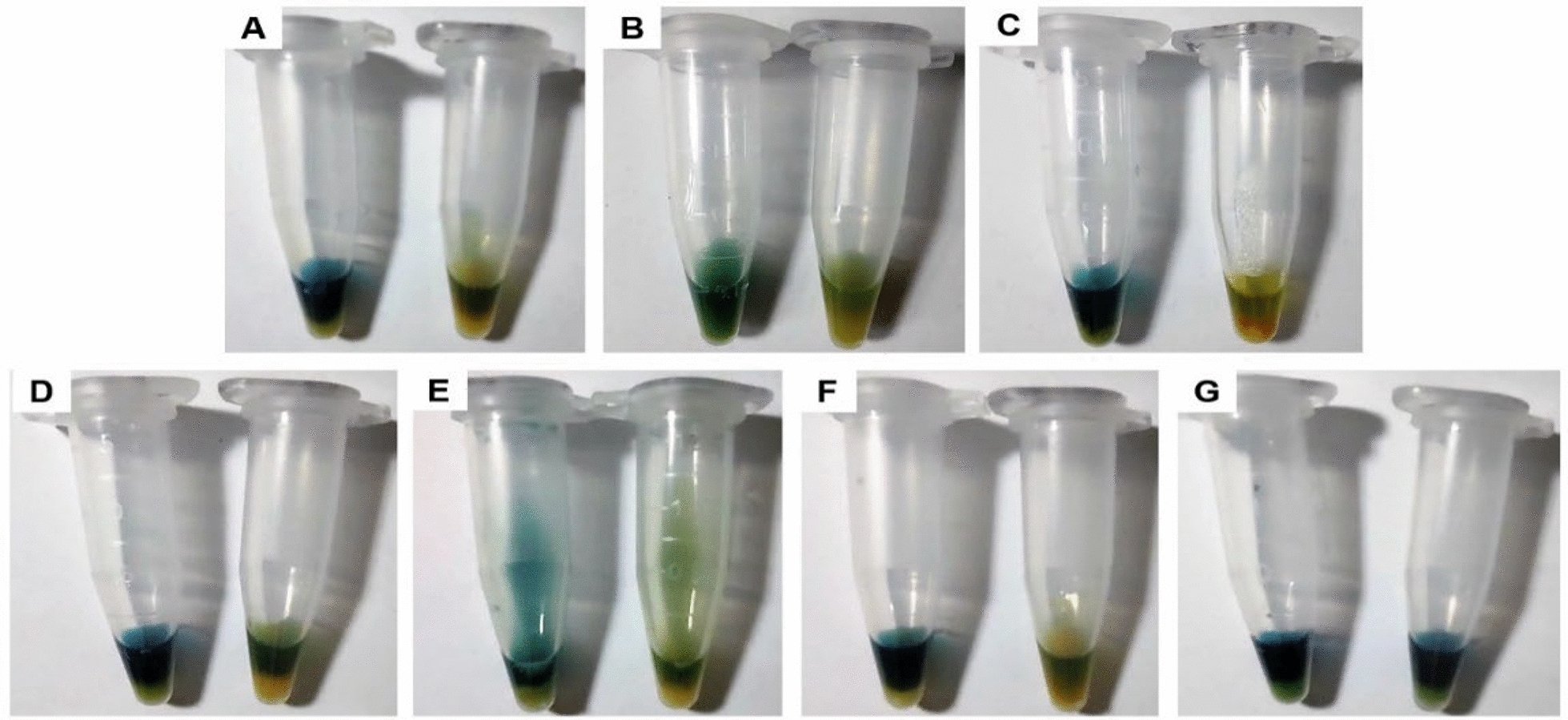
Carbapenemases production analyzed by Blue-Carba in influent (IF) strains growth in MCC + M plates. **a** *K. pasteurii* M2/A/C/34, **b** *K. pneumoniae* subsp. *pneumoniae* M3/A/M/3, **c** *C. freundii* s.str. M4/A/C/32, **d** *K. pneumoniae* UCO368 (KPC [+] control), **e**: *K. pneumoniae* UCO361 (NDM [ +] control), **f** *K. pneumoniae* UCO322 (OXA-48 [+] control), **g** *E. coli* ATCC25922 (negative control)

**Fig. 3:**
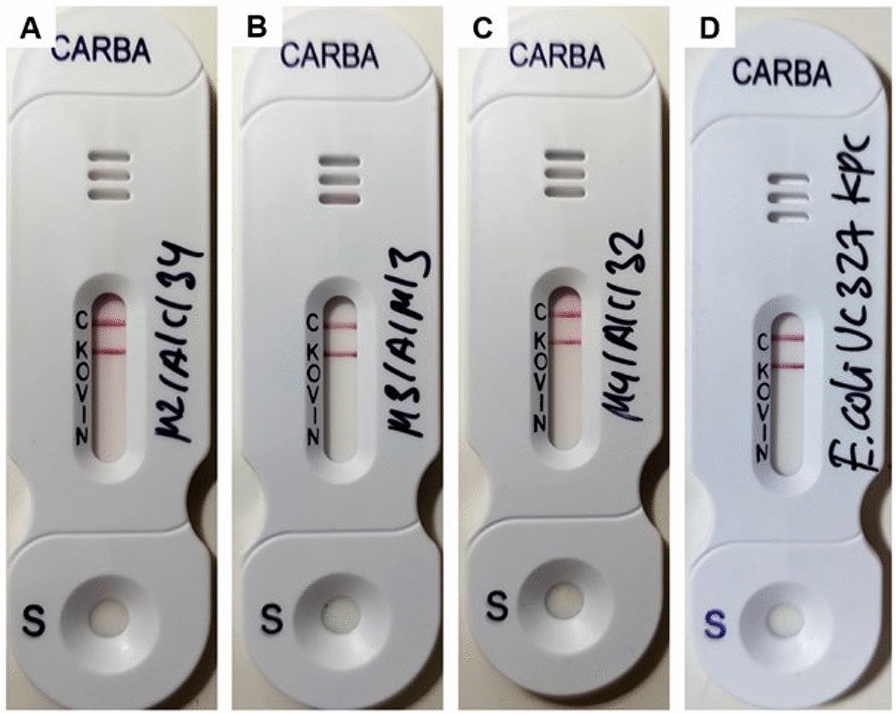
Carbapenemases identification by the NG-Test® CARBA-5 immunochromatographic test in carbapenem-resistant strains (N = 3) from IF samples. **a** *K. pasteurii* M2/A/C/34, **b** *K. pneumoniae* subsp. *pneumoniae* M3/A/M/3, **c** *C. freundii* s.str. M4/A/C/32, **d** *E. coli* UCO327 (KPC [+] control). C: control; K: KPC-*like*; O: OXA-48-*like*; V: VIM-*like*; I: IMP-*like*; N: NDM-*like*

All 3 isolates, designated as M2/A/C/34, M3/A/M/3, and M4/A/C/32, were identified through WGS data as *Klebsiella pasteurii*, *Klebsiella pneumoniae* subsp. *pneumoniae*, and *Citrobacter freundii* sensu stricto, respectively. These results were corroborated by ribosomal multilocus sequence typing (rMLST), average nucleotide identity by blast (ANIb), and digital DNA-DNA hybridization (DDH) analyses (Table S1). Moreover, these strains tested positive for carbapenemase production by the BlueCarba test (Fig. 2) and confirmed by the NG-Test^®^ CARBA-5 immunochromatographic cartridge, confirming the production of KPC-carbapenemases (Fig. 3). The immunochromatographic test results confirmed that the isolates were exclusively KPC producers, as they tested negative for all other carbapenemases included in the assay cartridge.

Antibiotic susceptibility testing revealed that the three carbapenemase-producing strains are not susceptible to nearly all tested β-lactam antibiotics (Table 1). Moreover, M4/A/C/32 isolate demonstrated susceptibility to CAZ, while M2/A/C/34 was susceptible to cefoxitin FOX. Among aminoglycosides, two isolates exhibited resistance or intermediate susceptibility to GEN and AMK, whereas one isolate was susceptible to both antibiotics (Table 1).

**Table 1:** Antibiotic susceptibility testing in carbapenemase-producing strains (N = 3) collected from influent (IF) samples

Strain	Antibiotics															
	FOX	CRO	CAZ	CTX	FEP	IPM	ETP	MEM	GEN	AMK	CIP	LVX	CHL	TET	SUL	TRI
*K. pasteurii* M2/A/C/34	S	R	R	R	SDD	I	I	R	S	S	R	S	S	S	S	S
*K. pneumoniae* subsp. *pneumoniae* M3/A/M/3	R	R	R	R	R	R	R	R	R	I	R	R	R	S	S	R
*C. freundii* s.str. M4/A/C/32	R	R	S	R	SDD	R	R	I	R	R	S	S	S	R	R	R

FOX, cefoxitin; CRO, ceftriaxone; CAZ, ceftazidime; CTX, cefotaxime; FEP, cefepime; IPM, imipenem; ETP, ertapenem; MEM, meropenem; GEN, gentamicin; AMK, amikacin; CIP, ciprofloxacin; LVX, levofloxacin; CHL, chloramphenicol; TET, tetracycline; SUL, compound sulfonamide; TRI, trimethoprim; S, susceptible; I, intermediate; R, resistant; SDD, dose-dependent susceptible

Furthermore, M3/A/M/3 isolate was resistant to a broader range of antibiotic classes compared to the other two isolates but remained susceptible to AMK, TET, and SUL. This was corroborated by the MAR index values, with M3/A/M/3 having a MAR index of 0.81 (Table 2). Based on the susceptibility test results, all three strains exhibited multidrug resistance (MDR) phenotypes [24]. Additionally, the MICs for IMP were 4, ≥ 32, and 4 µg/mL for M2/A/C/34, M3/A/M/3, and M4/A/C/32, respectively, in concordance with the disc diffusion results.

**Table 2:** Antibiotic resistance profiles in carbapenemase-producing strains

Strain	Antibiotype	Phenotype	MAR
*K. pasteurii* M2/A/C/34	CRO-CAZ-CTX-MEM-CIP	MDR	0.31
*K. pneumoniae* subsp. *pneumoniae* M3/A/M/3	FOX-CRO-CAZ-CTX-FEP-IPM-ETP-MEM-GEN-CIP-LVX-CHL-TRI	MDR	0.81
*C. freundii* s.str. M4/A/C/32	FOX-CRO-CTX-IPM-ETP-GEN-AMK-TET-SUL-TRI	MDR	0.63

MDR, multidrug-resistant; MAR, multiple antibiotic resistance index; FOX, cefoxitin; CRO, ceftriaxone; CAZ, ceftazidime; CTX, cefotaxime; FEP, cefepime; IPM, imipenem; ETP, ertapenem; MEM, meropenem; GEN, gentamicin; AMK, amikacin; CIP, ciprofloxacin; LVX, levofloxacin; CHL, chloramphenicol; TET, tetracycline; SUL, compound sulfonamide; TRI, trimethoprim

Analysis of the resistome revealed the presence of the *bla*_KPC-2_ variant in all three strains, along with multiple other β-lactamases-encoding genes. Specifically, five β-lactamases genes were identified in *K. pneumoniae* subsp. *pneumoniae* M3/A/M/3, three in *C. freundii* s.str. M4/A/C/32, and other three in *K. pasteurii* M2/A/C/34. Notably, *K. pasteurii* M2/A/C/34 harbored the extended-spectrum β-lactamase (ESBL) genes *bla*_CTX-M-177_ and *bla*_OXY-4–1_, while *C. freundii* s.str. M4/A/C/32 contained the AmpC β-lactamase gene *bla*_CMY-48_ (Table 3). Aminoglycoside-modifying enzyme (AME)-encoding genes were also detected in all isolates (Table 3). Furthermore, plasmids reconstruction and typing revealed that in all strains the *bla*_KPC-2_ gene was contained in plasmids. Specifically, this gene was carried by IncU, IncFII, and IncP6 plasmids identified in *K. pasteurii* M2/A/C/34, *K. pneumoniae* subsp. *pneumoniae* M3/A/M/3, and *C. freundii* s.str. M4/A/A/32, respectively (Table 3). Besides, the ARGs *bla*_TEM-1A_, *bla*_OXA-9_, *dfrA14*, *aph(6)-Id, aph(3″)Ib, sul1,* and *tet(B)* were also present in different plasmids harbored by these isolates (Table 3). Interestingly, *K. pneumoniae* subsp. *pneumoniae* M3/A/M/3 was found to harbor the fosfomycin resistance gene *fosA*, which is particularly relevant as this drug is being reintroduced into clinical practice.

**Table 3:** Genomic characteristics of the carbapenemase-producing strains recovered from influent (IF) samples (N = 3)

Strain	Species	ARGs	ST	Plasmids (MOB Type/Inc group)	In	VFGs	CS
M2/A/C/34	*K. pasteurii*	*bla*_CTX-M-177_; *bla*_OXY-4–1;_ ***bla***_KPC-2_*; *aac(6ʹ)-Ib-cr*; *fosA*	470	AA024 (NA/IncU)* AA055 (NA/NA) AA100 (NA/NA) AA121 (NA/NA) AA364 (NA/IncP) AA435 (NA/IncFII(pBK30683)) AA519 (NA/Col4401) AA882 (NA/NA) Novel (NA/IncFIB) Novel (MOB_F_/IncFII(pECLA))	*Intl1*	*ybt*	KL29 (U)
M3/A/M/3	*K. pneumoniae* subsp. *pneumoniae*	*bla*_SHV-11_; *bla*_TEM-1A_***; *bla*_OXA-1_; *bla*_OXA-9_***; ***bla***_KPC-2_*; *aac(3)-IIa*; *aac(6ʹ)-Ib-cr*; *fosA*; dfrA14**	273	AA019 (NA/IncFIB)*** AA028 (NA/Unk) AA103 (NA/ColRNAI) AA276 (NA/IncFIB) AA277 (NA/NA)** AA356 (NA/IncFII)* AA667 (NA/NA) AB531 (NA/rep_cluster_3) AB685 (NA/Col(MG828)) AE104 (NA/IncFII)	*Intl1*	-	KL74 (U)
M4/A/C/32	*C. freundii* s.str	*bla*_CMY-48*;*_* bla*_OXA-9_; ***bla***_KPC-2_*; aph(3ʺ)-Ib*; aph(6)-Id*; *aac(6ʹ)-Ib*; *aac(3)-Ia*; sul1**; tet(B)***; *dfrA1*	214	AA364 (MOB_P_/IncP6)* AA622 (MOB_H_/IncA) AB041 (MOB_H_/Col440I) AB130 (MOB_H_/RepA (pKPC-CAV1321))** AF098 (NA/NA)*** Novel (NA/rep_cluster_1195)	–	*chuX* *ompA* *fliG* *csgB* *csgD* *csgE* *csgF* *csgG*	–

ARGs: antibiotic resistance genes, ST: sequence type, In: integron, VFGs: virulence factors genes, CS: capsular serotype (k-locus), NA: not assigned. The *bla*_KPC_-variants of the strains under study are indicated in bold. Plasmid typing is indicated between parenthesis according to the prediction performed by MOB Suite and PlasmidFinder tools. The search for CS is not available for *C. freundii* in the Kaptive database. (U) means that the CS of the strains are unknown, however they are close to those indicated in the table. Asterisks in the ARGs and plasmids names indicate their association

The MAR index results indicate that *K. pneumoniae* subsp. *pneumoniae* M3/A/M/3 exhibited the highest value (0.81), followed by *C. freundii* s.str. M4/A/C/32 (0.63), and *K. pasteurii* M2/A/C/34 (0.31) (Table 2).

MLST analysis revealed that *K. pasteurii* M2/A/C/32, *K. pneumoniae* subsp. *pneumoniae* M3/A/M/3, and *C. freundii* s.str. M4/A/C/32 belonged to ST470, ST273, and ST214, respectively (Table 3). Additionally, MOB-Suite and PlasmidFinder results showed that *K. pasteurii* M2/A/C/34 and *K. pneumoniae* subsp. *pneumoniae* M3/A/M/3 harbored ten plasmids, whereas *C. freundii* s.str. M4/A/C/32 carried six plasmids (Table 3). Notably, the majority of these plasmids belonged to the IncF and IncP groups, which are distinguished for their broad host range across *Enterobacterales* species and even *P. aeruginosa* [56] (Table 3). Moreover, those plasmids that were not typeable by MOB suite (Table 3) were classified as non-mobilizable.

Furthermore, both *K. pasteurii* M2/A/C/34 and *K. pneumoniae* subsp. *pneumoniae* M3/A/M/3 harbored a class 1 integron element (Table 3). Moreover, *K. pasteurii* M2/A/C/34 also carried a yersiniabactin (*ybt*) gene, which is associated with iron acquisition and virulence. Investigation of capsular serotypes (CSs) identified unknown *k*-loci closely related to KL29 and KL74 in *K. pasteurii* M2/A/C/34 and *K. pneumoniae* subsp. *pneumoniae* M3/A/M/3, respectively (Table 3). We were unable to determine the CS for *C. freundii* s.str M4/A/C/32 using Kaptive, as this species is non-capsulated. However, a search in the VFDB identified eight virulence factor genes (VFGs) in *C. freundii* s.str M4/A/C/32. These included genes encoding a heme-binding protein (*chuX*), outer membrane protein A (*ompA*), flagellar motor switch protein G (*fliG*), and different curli proteins (*csgB/D/E/F/G*) (Table 3). As such, *C. freundii* s.str M4/A/C/32 exhibited the most extensive virulome and resistome among the three strains analyzed.

**Fig. 4:**
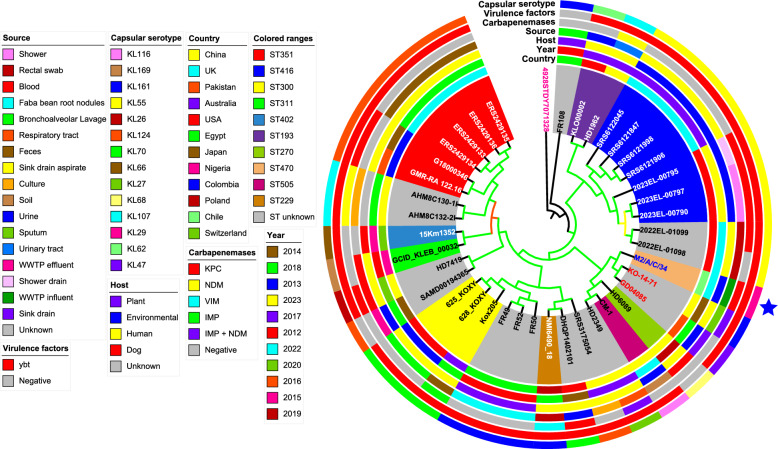
Maximum likelihood phylogenomic tree depicting the relationship among *K. pasteurii* M2/A/C/34 and 38 other strains of the same species from around the world. The phylogeny was inferred in IQ-TREE v2.1.2 from core-genomes aligned by Roary v3.13.0 and filtered by SNP-sites v2.5.1. The values of the SH-aLRT, aBayes and UFboot are indicated in colors at the branches, which were performed based on 1000 replicates. The orange, yellow and green branches represent values between [70–80], [81–90] and ≥ 91 for SH-aLRT and UFBoot respectively, and [0.7–0.8], [0.81–0.9] and ≥ 0.91 for aBayes respectively. The colored ranges represent the strains STs. The strain M2/A/C/34 is labeled in bold blue and indicated by blue star while the close phylogenomically strains are in bold red. The tree was annotated and viewed in ITOL v6.8.2 and *Klebsiella grimontii* 4928STDY7071328 labeled in bold purple was used as outgroup. Branch lengths are not to scale, and the strains GenBank access numbers are found in the Table S2

In the phylogenomic analysis of *K. pasteurii* M2/A/C/34, a total of 40 taxa were examined, including this isolate and 38 additional genomes of the same species from various geographic locations, with a *K. grimontii* genome serving as the outgroup. The results showed that *K. pasteurii* M2/A/C/34 is closely related to strains KO-14-71 and GD0485, which were isolated in China and Pakistan in 2014 and 2018, respectively (Fig. 4). These strains were recovered from human sputum and a sink drain, respectively, but have not yet been assigned a ST. Furthermore, we found that the majority of *K. pasteurii* genomes are not assigned to specific STs, with ST416 and ST351 being the most frequently identified among those with assigned STs. Most *K. pasteurii* genomes originated from isolates collected between 2017 and 2018, primarily from human samples in the UK, USA, and China. Interestingly, the majority of *K. pasteurii* genomes do not carry carbapenemase genes. Among the carbapenemase-positive isolates, the *bla*_KPC_ gene was the most prevalent (Fig. 4 and Table S2). Additionally, most genomes were found to harbor the *ybt* virulence gene cluster, which is widely distributed among the *Klebsiella* genus [57] (Fig. 4 and Table S2).

**Fig. 5:**
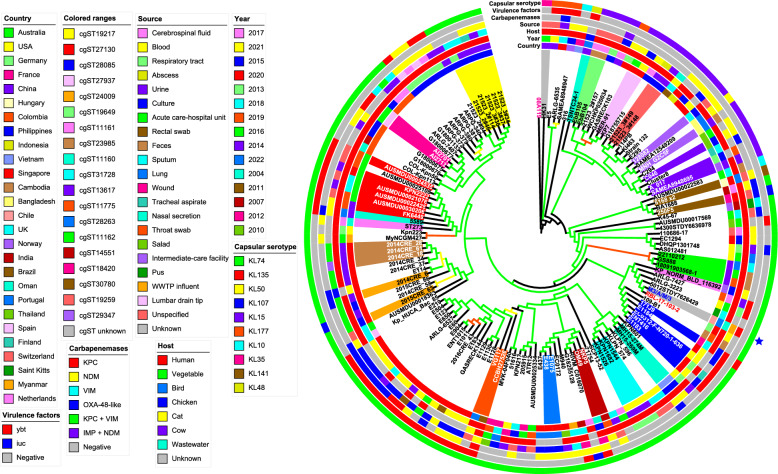
Maximum likelihood phylogenomic tree depicting the relationship among *K. pneumoniae* subsp. *pneumoniae* M3/A/M/3 ST273 and 138 other strains of the same species and sequence types (STs) from around the world. The phylogeny was inferred in IQ-TREE v2.1.2 from core-genomes aligned by Roary v3.13.0 and filtered by SNP-sites v2.5.1. The values of the SH-aLRT, aBayes and UFboot are indicated in colors at the branches, which were performed based on 1000 replicates. The orange, yellow and green branches represent values between [70–80], [81–90] and ≥ 91 for SH-aLRT and UFBoot respectively, and [0.7–0.8], [0.81–0.9] and ≥ 0.91 for aBayes respectively. The colored ranges represent the most frequent core-genomes sequence types (cgSTs) of the strains and the others are found in the Table S3. The strain M3/A/M/3 is labeled in bold blue and indicated by blue star while the close phylogenomically strain are in bold red. The tree was annotated and viewed in ITOL v6.8.2 and *Klebsiella quasivariicola* 08A119 labeled in bold purple was used as outgroup. Branch lengths are not to scale, and the strains GenBank access numbers are found in the Table S3

In our phylogenomic analysis of *K. pneumoniae* subsp. *pneumoniae* M3/A/M/3, we examined a dataset of 140 taxa, including 138 genomes of the same species from ST273 collected globally, using a *K. quasivariicola* genome as an outgroup. The analysis indicated that M3/A/M/3 is closely related to the RBL-17-103-2 isolate, which was recovered from human blood in Germany in 2020. Most of the analysed genomes originated from human samples collected in Singapore, China, the Philippines, Colombia, Australia, and the USA, primarily between 2014 and 2021. These isolates exhibited a variety of carbapenemase genes, with *bla*_KPC_, *bla*_NDM_, and *bla*_OXA-48-like_ being the most frequently detected. Furthermore, the *ybt* virulence gene cluster emerged as the most common virulence factor group among the ST273 isolates (Fig. 5 and Table S3).

Regarding CSs, the predominant types identified among ST273 isolates were KL74 and KL15, with *K. pneumoniae* subsp. *pneumoniae* M3/A/M/3 primarily associated with KL74 (Fig. 5 and Table S3). In the case of *C. freundii* s.str. M4/A/C/32, the closest genome identified by the GTDB was *C. freundii* ATCC 8090 (GCF_011064845.1).

## Discussion

In this study, we report the first detection of KPC-producing carbapenem-resistant *Enterobacterales* in WW from the metropolitan area of Gran Concepción, Chile. In terms of antibiotics usage, Allel et al. demonstrated that Hospitals located in the Gran Concepcion Metropolitan Area had one of the highest rates in Chile during the 2008–2017 period, especially in the usage of methicillin, imipenem, meropenem, piperacillin/tazobactam and vancomycin [58]. This factor could be producing a selective pressure in the local community, facilitating the emergence and spread of resistant bacteria, including those in WWTP. In this sense, among the isolates included in this study, all displayed MAR indexes that represent a risk of contamination. Specifically, this index serves as an effective tool for monitoring antibiotic-resistant organisms and evaluating the associated risk. A MAR value greater than 0.2 specifically suggests that the contamination source is likely an environment with frequent antibiotic usage [59]. Accordingly, all strains exhibited MAR indexes > 0.2, thus representing a high risk of contamination where antibiotics are frequently used [60].

Interestingly, antibiotic susceptibility testing revealed that all three carbapenemase-producing strains exhibited non-susceptibility (resistant or intermediate) to most β-lactam antibiotics tested, with the exceptions of M4/A/C/32, which retained susceptibility to CAZ, and M2/A/C/34, which was susceptible to FOX. These findings provide critical insights into the resistomes and phenotypes of carbapenem-resistant *Enterobacterales* in environmental reservoirs.

In this context, the presence of *bla*_KPC-2_ in all three strains aligns with previous studies that have detected carbapenemase-producing Gram-negative bacilli in WWTP. For instance, a study from Finland reported 59 isolates in WW samples of 1L collected between October 2011 and August 2012, with 6 of these carrying *bla*_KPC_ [61]. Similarly, research conducted in Spain identified 252 carbapenemases-producing Gram negative isolates in WW samples collected between April 2020 and February 2022, 162 of which harboured *bla*_KPC_ [62]. Interestingly, Further WGS analysis of 55 of these strains revealed that 34 carried the *bla*_KPC-2_ variant [62]. However, it is important to consider that these studies differ in the initial volume of WW sampled, as the work from Finland used 1L, similar to our study, whereas the study from Spain utilized 500 mL, thus the prevalence of resistant bacteria should consider this factor.

Additionally, Khavandi et al. found 17% of *Enterobacterales* resistant to carbapenems in municipal wastewater in Iran [63], where *bla*_OXA-48_ gene was the predominant among these isolates (97%). Moreover, Hoelle et al. recovered 322 *E. coli* isolates from seven WWTPs from the USA, of which 65 were resistant to imipenem, being *bla*_VIM_ the predominant carbapenemase gene [64].

In the context of South America, Rodríguez et al. reported the presence of KPC-producing *Klebsiella pneumoniae* in WWTPs in Colombia, where carbapenem-resistant Gram-negative isolates accounted for 38.2% (n = 149) of the total isolates recovered (n = 390) [65]. These findings underscore the extent of environmental contamination concerning these multidrug-resistant pathogens, especially those resistant to carbapenems. In contrast, our findings revealed the presence of three carbapenem-resistant Gram-negative isolates out of a total of 205, representing a prevalence of 1.5%, which is significantly lower than other studies described previously.

Notably, our study did not detect co-occurrences of *bla*_KPC_ with other carbapenemases, which contrasts with findings reported by Wang et al*.* [66]. In their study, co-occurrences of *bla*_KPC_ with *bla*_NDM_ and *bla*_OXA-48-like_ genes were identified in carbapenemase-producing Gram-negative bacilli isolated from WW in urban environments. Such co-occurrences are indicative of enhanced genetic exchange among resistant bacteria, facilitated by mobile genetic elements like plasmids and integrons [66]. Similarly, another study identified 15 carbapenemase-producing *K. pneumoniae* strains resistant to carbapenems in WW samples from three WWTPs in Brazil [67]. These strains harboured diverse carbapenemase genes, including *bla*_KPC_, *bla*_NDM_, and *bla*_OXA-48-like_, and exhibited high resistance rates to multiple antibiotics, underscoring the role of WWTPs as hotspots for AMR dissemination [67]. The absence of co-occurring carbapenemases in our isolates may suggest regional differences in the genetic composition of carbapenem-resistant *Enterobacterales* or variations in the selective pressures present in WW environments in Chile.

Furthermore, we determined that strain M2/A/C/34 exhibited sensitivity to GEN and AMK. This sensitivity could be attributed to the presence of the *aac(6ʹ)-Ib-cr* gene, which confers resistance to quinolones at varying levels [57, 58], as well as tobramycin and kanamycin, but not to GEN [59, 60]. However, as noted by phenotypic resistance to AMK could be not fully expressed, which can affect its acetylating capacity against this antibiotic [61, 62].

Based on the MLST analysis, *K. pasteurii* M2/A/C/32, *K. pneumoniae* subsp. *pneumoniae* M3/A/M/3, and *C. freundii* s.str M4/A/C/32 were assigned to STs ST470, ST273 and ST214, respectively, which represents the first documentation of these STs in Chile. Specifically, ST273 is globally distributed, as confirmed by our phylogenomic analysis of *K. pneumoniae* subsp. *pneumoniae* M3/A/M/3. In contrast, ST470 has only been previously described in a *K. pasteurii* strain isolated from cattle in Japan in 2014 [74]. Similarly, ST214 has been identified in two *Citrobacter* sp. strains isolated from humans and food in China in 2015 and 2017, respectively [74].

On the other hand, the predominant plasmids identified in the strains belong to the IncF and IncP groups. Remarkably, the IncFIB type plasmid detected in *K. pasteurii* M2/A/C/32 and in *K. pneumoniae* subsp. *pneumoniae* M3/A/M/3 has been previously associated with ESBL genes such as *bla*_CTX-M-1_, and *bla*_CTX-M-15_ [64–66], and with carbapenemases genes such as *bla*_KPC-2_, *bla*_NDM-1_ [67–69]_._ Additionally, IncP was detected in *K. pasteurii* M2/A/C/34 and *C. freundii* s.str M4/A/C/32 strains, which has been primarily associated with *bla*_KPC-2_ [55, 70–72]. These findings underscore the impact of specific plasmids types in the spread of carbapenemases and ESBLs genes of clinical relevance.

Furthermore, *C. freundii* M4/A/C/32 exhibited the most extensive resistome and virulome among the strains analysed. This finding is particularly intriguing, as *Citrobacter* species are typically considered low-virulence, nosocomial pathogens that are often susceptible to carbapenems, aminoglycosides, and tetracyclines [84]. In contrast, other authors reported a *C. europaeus* strain isolated from ascitic fluid that exhibited high resistant and virulent characteristics similar to those of our strain [85]. This strain harbored the carbapenemase and ESBL genes *bla*_NDM-1_, *bla*_SHV-12_ respectively, along with several AME genes with acetylating and phosphorylating activity. Additionally, its virulome included genes comparable to those detected in our strain, along with additional virulence-associated genes [85].

Moreover, the *K. pasteurii* M2/A/C/34 strain is genetically related with strains *K. pasteurii* KO-14-71 and *K. pasteurii* GD0485, forming a primary clade among these three bacteria. However, M2/A/C/34 differs in 7614 SNPs with KO-14-71 and 7629 SNPs with GD0485, respectively (Figure S1). Additionally, the strain *K. pneumoniae* subsp. *pneumoniae* M3/A/M/3 was clustered with RBL-17-103-2 strain, configurating a monophyletic group. However, M3/A/M/3 has 106 SNPs of difference with RBL-17-103-2 (Figure S2). These results suggest that strain RBL-17-103-2 and M3/A/M/3 have a similar origin, in which the latter acquired the *bla*_KPC-2_ gene through horizontal transfer from a local bacterium carrying this ARG, as the German strain did not possess *bla*_KPC_ or any other carbapenemase gene (Fig. 5 and Table S3) [75–77]. In this sense, Schürch et al. established a threshold of <18 SNPs to determine genetic relatedness among *Klebsiella* genera [86], our strains displayed greater SNPs values compared to the threshold described above, evidencing that our strains are not closely related with others from the phylogenetic tree.

## Conclusions

Our study corresponds to the first identification of CRE species carrying *bla*_KPC-2_ in WW samples from the Gran Concepción Metropolitan area, Chile. Among these, *K. pasteurii* had not been previously reported in the country. Furthermore, the isolated found in this work belong to STs (ST470, ST273, and ST214), which had not been previously reported in Chile. We did not detect co-carriage of other carbapenemase genes in our bacteria, but we primarily found co-carriage with ESBLs and AMEs genes. As such, the isolates exhibited MDR profiles, in which *K. pneumoniae* subsp. *pneumoniae* M3/A/M/3 possessed the broadest resistance profile, as well as the highest MAR index and MIC values for IPM. On the other hand, *C. freundii* s.str M4/A/C/32 showed the largest virulome among the strains. Phylogenomic analyses of *K. pasteurii* M2/A/C/34 and *K. pneumoniae* subsp. *pneumoniae* M3/A/M/3 strains allowed us to infer that the emergence of the former was due to the introduction of an ancestral foreign strain from Asia between 2014 and 2022. Meanwhile, the latter was likely transported from Germany to Chile between 2020 and 2022, subsequently acquiring the *bla*_KPC-2_ gene in Chile from a local strain carrying this ARG. However, we were unable to construct a phylogenomic tree for the *C. freundii* s.str M4/A/C/32 strain due to the absence of genomes belonging to ST214. The results of this study underscore the importance of conducting genomic surveillance in wastewater under the One Health approach, which is crucial for understanding the impact of disseminating bacteria carrying KPC and other carbapenemases.

## Supplementary Information

Additional file 1.

Additional file 2.

Additional file 3.

Additional file 4.

Additional file 5.

## Data Availability

This Whole Genome Shotgun project has been deposited at DDBJ/ENA/GenBank under the accession numbers JBHJCW000000000.1 (M2/A/C/34), JBHJCX000000000.1 (M3/A/M/3), and JBHJCV000000000.1 (M4/A/C/32).
