# Supplementary material for: Detection of KPC-producing Enterobacterales species in wastewater samples from the Gran Concepción Metropolitan area, Chile
Source: Biol Res. 2025 Jun 7;58:35. doi: 10.1186/s40659-025-00612-7 (PMC12144836; doi:10.1186/s40659-025-00612-7)
Supplement: Supplementary file 1 — Additional file 1. [file 40659_2025_612_MOESM1_ESM.docx]

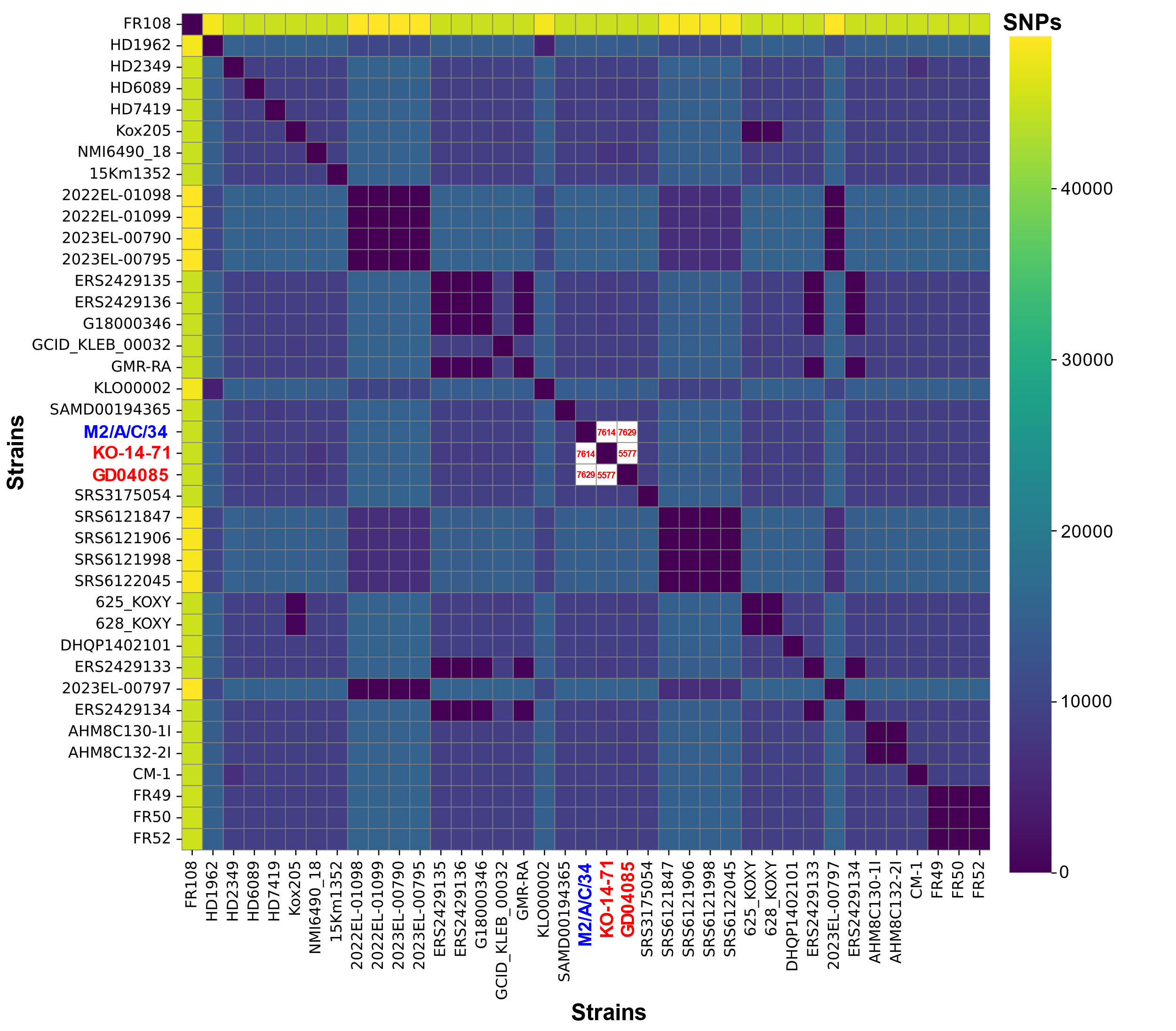


**Figure S1. SNPs Distance Matrix depicting the relationship among *K. pasteurii* M2/A/C/34 and 38 other strains of the same species from around the world.** The strain M2/A/C/34 is labeled in bold blue while the close phylogenomically strains KO-14-71 and GD04085 are in bold red. In the center of the figure, the SNPs values between the three mentioned strains are indicated in bold red.
