# Supplementary material for: Detection of KPC-producing Enterobacterales species in wastewater samples from the Gran Concepción Metropolitan area, Chile
Source: Biol Res. 2025 Jun 7;58:35. doi: 10.1186/s40659-025-00612-7 (PMC12144836; doi:10.1186/s40659-025-00612-7)
Supplement: Supplementary file 2 — Additional file 2. [file 40659_2025_612_MOESM2_ESM.docx]

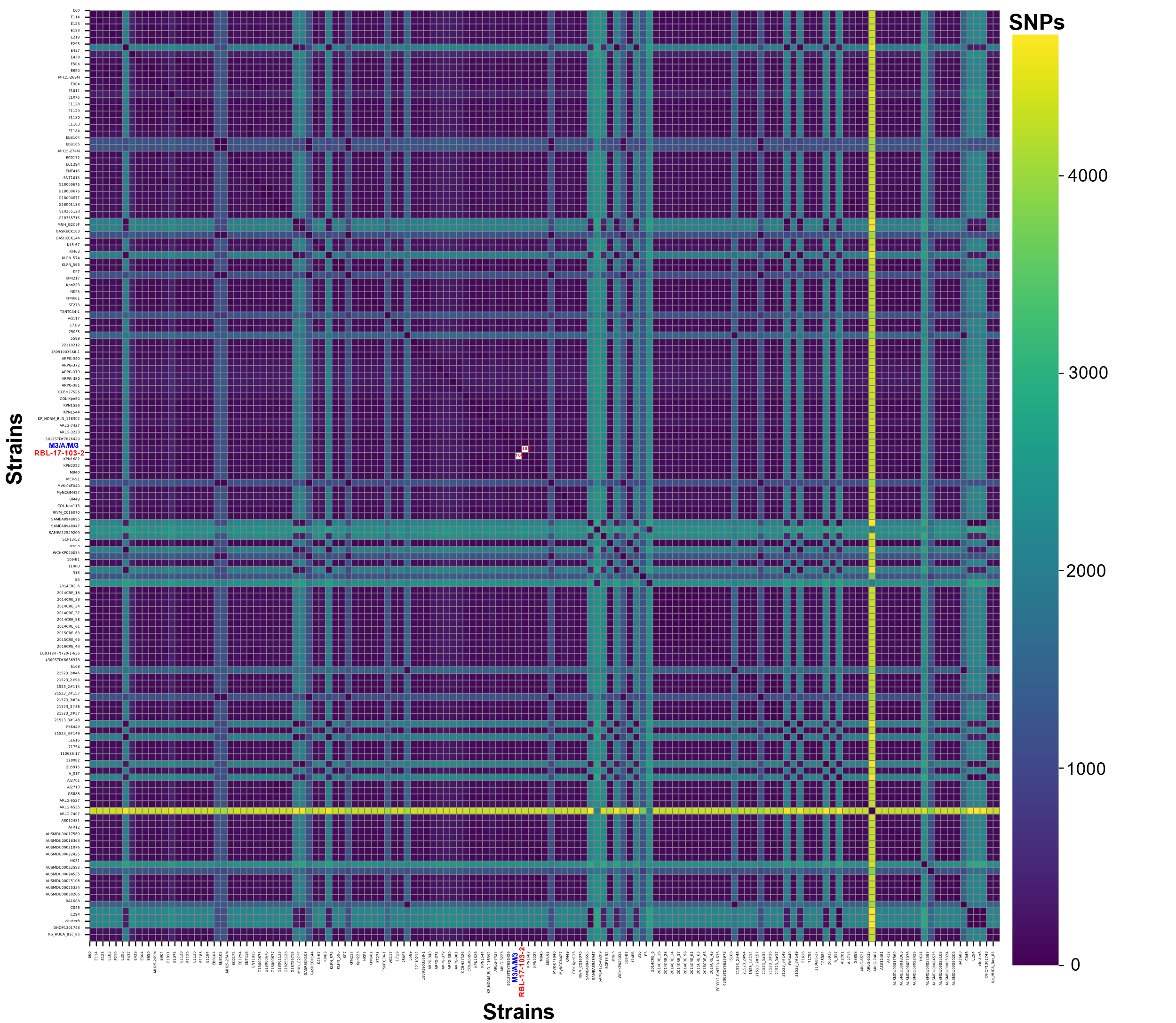


**Figure S2. SNPs Distance Matrix depicting the relationship among** ***K. pneumoniae* subsp. *pneumoniae* M3/A/M/3 ST273 and 138 other strains of the same species and sequence types (STs) from around the world.** The strain M3/A/M/3 is labeled in bold blue while the close phylogenomically strain RBL-17-103-2 is in bold red. In the center of the figure, the 106 SNPs of difference between the two mentioned strains are indicated in bold red.
