# Supplementary material for: Detection of KPC-producing Enterobacterales species in wastewater samples from the Gran Concepción Metropolitan area, Chile
Source: Biol Res. 2025 Jun 7;58:35. doi: 10.1186/s40659-025-00612-7 (PMC12144836; doi:10.1186/s40659-025-00612-7)
Supplement: Supplementary file 3 — Additional file 3. [file 40659_2025_612_MOESM3_ESM.docx]

**Table S1.** Sequencing statistics and genomic identification of the carbapenemase-producing strains recovered from influent (IF) samples (N=3).

| Strain name | Sequencing Statistics | | | | | | | | | | Genomic identification | | |
| --- | --- | --- | --- | --- | --- | --- | --- | --- | --- | --- | --- | --- | --- |
|  | **COV** | **NC** | **N50** | **TL (bp)** | **GC (%)** | **C (%)** | **S (%)** | **D (%)** | **F (%)** | **M (%)** | **Reference strain** | **ANIb (%)** | **DDH (%)** |
| M2/A/C/34  (GCA_042159875.1) | 100x | 261 | 123451 | 6353698 | 55,2 | 98,6 | 98,2 | 0,5 | 0,5 | 0,9 | *K. pasteurii* (GCA_018139045.1) | 99,27 | 97,90 |
|  |  |  |  |  |  |  |  |  |  |  | *K. grimontii* (GCA_902164675.1) | 95,97 | 70,60 |
|  |  |  |  |  |  |  |  |  |  |  | *K. michiganensis* (GCA_015139575.1) | 93,75 | 57,60 |
|  |  |  |  |  |  |  |  |  |  |  | *K. oxytoca* (GCA_003812925.1) | 91,15 | 47,10 |
| M3/A/M/3  (GCA_042159955.1) | 100x | 233 | 247839 | 5663140 | 57,1 | 98,4 | 98,0 | 0,5 | 0,5 | 1,1 | *K. pneumoniae* subsp. *pneumoniae* (GCA_000240185.2) | 98,58 | 93,90 |
|  |  |  |  |  |  |  |  |  |  |  | *K. pneumoniae* subsp. *rhinoscleromatis* (GCA_000163455.1) | 97,96 | 92,00 |
|  |  |  |  |  |  |  |  |  |  |  | *K. pneumoniae* subsp. *ozaenae* (GCA_001598715.1) | 97,94 | 93,10 |
|  |  |  |  |  |  |  |  |  |  |  | *K. variicola* (GCA_009648975.1) | 94,24 | 60,80 |
|  |  |  |  |  |  |  |  |  |  |  | *K. variicola* subsp. *variicola* (GCA_020525545.1) | 94,24 | 58,60 |
|  |  |  |  |  |  |  |  |  |  |  | *K. variicola* subsp. *tropica* (GCA_026802135.1) | 93,85 | 60,90 |
|  |  |  |  |  |  |  |  |  |  |  | *K. quasipneumoniae* KqPF26 (GCA_016415705.1) | 93,54 | 57,50 |
|  |  |  |  |  |  |  |  |  |  |  | *K. quasipneumoniae* subsp. *quasipneumoniae* (GCA_020525925.1) | 93,48 | 57,10 |
|  |  |  |  |  |  |  |  |  |  |  | *K. quasipneumoniae* subsp. *similipneumoniae* (GCA_003181175.1) | 93,64 | 57,50 |
| M4/A/C/32  (GCA_042159915.1) | 100x | 159 | 97556 | 5271610 | 51,4 | 99,5 | 99,1 | 0,5 | 0,2 | 0,2 | *C. freundii* (GCA_003812345.1) | 98,51 | 92,10 |
|  |  |  |  |  |  |  |  |  |  |  | *C. portucalensis* (GCA_008693605.1) | 94,44 | 60,50 |
|  |  |  |  |  |  |  |  |  |  |  | *C. braakii* (GCA_009648935.1) | 92,44 | 50,60 |
|  |  |  |  |  |  |  |  |  |  |  | *C. youngae* (GCA_030294585.1) | 90,61 | 47,00 |

**COV:** Coverage depth, **NC:** Number of contigs, **TL (bp):** Total length in bp, **C (%):** Completeness, **S (%):** Percentage of complete and single-copy genes, **D (%):** Percentage of complete and duplicated genes, **F (%):** Percentage of fragmented genes, **M (%):** Percentage of missing genes, **ANIb (%):** Average nucleotide identity, **DDH (%):** DNA-DNA hybridization percentage *in silico*. Reference species with the highest ANIb (%) and DDH (%) values are indicated in red, and the corresponding GenBank accession number is indicated next to each species. The three strains had a 100% identity value in rMLST against the highlighted species in red.
