# Supplementary material for: Detection of KPC-producing Enterobacterales species in wastewater samples from the Gran Concepción Metropolitan area, Chile
Source: Biol Res. 2025 Jun 7;58:35. doi: 10.1186/s40659-025-00612-7 (PMC12144836; doi:10.1186/s40659-025-00612-7)
Supplement: Supplementary file 4 — Additional file 4. [file 40659_2025_612_MOESM4_ESM.docx]

**Table S2.** Genomic characteristics of the strains employed in the *K. pasteurii* M2/A/C/34 phylogenomic tree.

| **Strain** | **Access number** | **Year** | **Country** | **Host** | **Source** | **ST** | **CGs** | **VFGs** | **CS** |
| --- | --- | --- | --- | --- | --- | --- | --- | --- | --- |
| M2/A/C/34 | GCA_042159875.1 | 2022 | Chile | Environmental | WWTP influent | 470 | *bla*_KPC_ | *ybt* | KL29 |
| FR52 | JARANL000000000.1 | 2012 | Egypt | Plant | Faba bean root nodules | Unknown | Negative | *ybt* | KL16 |
| FR49 | JARANN000000000.1 | 2012 | Egypt | Plant | Faba bean root nodules | Unknown | Negative | *ybt* | KL161 |
| FR50 | JARANM000000000.1 | 2012 | Egypt | Plant | Faba bean root nodules | Unknown | Negative | *ybt* | KL161 |
| 625_KOXY | SRR1656057 | 2013 | USA | Human | Bronchoalveolar Lavage | 300 | Negative | *ybt* | KL70 |
| 628_KOXY | SRR1656060 | 2013 | USA | Human | Bronchoalveolar Lavage | 300 | Negative | *ybt* | KL70 |
| Kox205 | GCA_021228735.1 | 2018 | Australia | Human | Feces | 300 | *bla*_IMP_ | *ybt* | KL70 |
| KO-14-71 | GCA_019661035.1 | 2014 | China | Human | Sputum | Unknown | *bla*_IMP_ *bla*_NDM_ | *ybt* | KL161 |
| GD04085 | JAOEBM000000000.1 | 2018 | Pakistan | Environmental | Sink drain | Unknown | Negative | *ybt* | KL47 |
| AHM8C130-1I | GCA_030169945.1 | 2018 | China | Unknown | Culture | Unknown | *bla*_NDM_ | *ybt* | KL107 |
| AHM8C132-2I | JANGSN000000000.1 | 2018 | China | Unknown | Culture | Unknown | *bla*_NDM_ | *ybt* | KL107 |
| 15Km1352 | JAPJJB000000000.1 | 2015 | Switzerland | Dog | Unknown | 402 | Negative | *ybt* | KL66 |
| SRS3175054 | SRR7012022 | 2017 | China | Human | Culture | Unknown | Negative | *ybt* | KL124 |
| SAMD00194365 | DRR199201 | 2018 | Japan | Environmental | WWTP effluent | Unknown | Negative | Negative | KL124 |
| ERS2429133 | ERR3403048 | 2018 | UK | Human | Feces | 351 | Negative | *ybt* | KL124 |
| ERS2429134 | ERR3403049 | 2018 | UK | Human | Feces | 351 | Negative | *ybt* | KL124 |
| ERS2429135 | ERR3403050 | 2018 | UK | Human | Feces | 351 | Negative | *ybt* | KL124 |
| ERS2429136 | ERR3403051 | 2018 | UK | Human | Feces | 351 | Negative | *ybt* | KL124 |
| GMR-RA 122.16 | ERR4968645 | 2016 | Colombia | Human | Unknown | 351 | *bla*_KPC_ | *ybt* | KL124 |
| G18000346 | ERR4968658 | 2014 | Colombia | Human | Unknown | 351 | *bla*_KPC_ | *ybt* | KL124 |
| GCID_KLEB_00032 | SRR5514213 | 2012 | Nigeria | Human | Blood | 311 | Negative | *ybt* | KL169 |
| HD2349 | JAQSKV000000000.1 | 2017 | China | Human | Respiratory tract | Unknown | *bla*_IMP_ *bla*_NDM_ | *ybt* | KL27 |
| CM-1 | GCA_031593235.1 | 2022 | China | Environmental | Soil | 505 | Negative | *ybt* | KL116 |
| HD7419 | JAQSLB000000000.1 | 2020 | China | Human | Blood | Unknown | *bla*_KPC_ | *ybt* | KL26 |
| HD6089 | JAQSLD000000000.1 | 2019 | China | Human | Blood | 270 | Negative | Negative | KL68 |
| NMI6490_18 | JATAUJ000000000.1 | 2018 | Poland | Human | Rectal swab | 229 | *bla*_VIM_ | *ybt* | KL161 |
| DHQP1402101 | SRR11193633 | 2014 | USA | Human | Urine | Unknown | *bla*_KPC_ | *ybt* | KL70 |
| 2022EL-01098 | JAQNDI000000000.1 | 2022 | USA | Human | Rectal swab | Unknown | Negative | *ybt* | KL55 |
| 2022EL-01099 | JAQNDH000000000.1 | 2022 | USA | Human | Rectal swab | Unknown | *bla*_KPC_ | *ybt* | KL55 |
| 2023EL-00790 | JAVDKU000000000.1 | 2023 | USA | Environmental | Shower | 416 | *bla*_KPC_ | *ybt* | KL55 |
| 2023EL-00797 | JAVDLB000000000.1 | 2023 | USA | Environmental | Shower | 416 | *bla*_KPC_ | *ybt* | KL55 |
| 2023EL-00795 | JAVDKZ000000000.1 | 2023 | USA | Environmental | Shower drain | 416 | *bla*_KPC_ | *ybt* | KL55 |
| SRS6121906 | SRR11046373 | 2017 | UK | Environmental | Sink drain aspirate | 416 | Negative | *ybt* | KL55 |
| SRS6121847 | SRR11046432 | 2017 | UK | Environmental | Sink drain aspirate | 416 | Negative | *ybt* | KL55 |
| SRS6122045 | SRR11046699 | 2017 | UK | Environmental | Sink drain aspirate | 416 | Negative | *ybt* | KL55 |
| SRS6121998 | SRR11046747 | 2017 | UK | Environmental | Sink drain aspirate | 416 | Negative | *ybt* | KL55 |
| HD1962 | JAQSLI000000000.1 | 2017 | China | Human | Urinary tract | 193 | *bla*_NDM_ | *ybt* | KL107 |
| KLO00002 | SRR7297487 | 2017 | USA | Human | Urine | 193 | Negative | *ybt* | KL62 |
| FR108 | JARANK000000000.1 | 2012 | Egypt | Plant | Faba bean root nodules | Unknown | Negative | NF | KL161 |

**ST**: sequence-type. **CGs**: carbapenemases genes. **VFGs**: virulence-factors genes. **CS**: capsular serotype (k-locus).
