# Supplementary material for: Detection of KPC-producing Enterobacterales species in wastewater samples from the Gran Concepción Metropolitan area, Chile
Source: Biol Res. 2025 Jun 7;58:35. doi: 10.1186/s40659-025-00612-7 (PMC12144836; doi:10.1186/s40659-025-00612-7)
Supplement: Supplementary file 5 — Additional file 5. [file 40659_2025_612_MOESM5_ESM.docx]

**Table S3.** Genomic characteristics of ST273 strains employed in the *K. pneumoniae* subsp. *pneumoniae* M2/A/C/34 phylogenomic tree.

| **Strain** | **Access number** | **Year** | **Country** | **Host** | **Source** | **cgST** | **CGs** | **VFGs** | **CS** |
| --- | --- | --- | --- | --- | --- | --- | --- | --- | --- |
| M3/A/M/3 | GCA_042159955.1 | 2022 | Chile | Environmental | WWTP influent | Unknown | *bla*_KPC_ | Negative | KL74 |
| ENT1015 | SRR9217864 | 2014 | Singapore | Human | Rectal swab | **34322** | *bla*_KPC_ | *ybt* | KL74 |
| E904 | SRR15695502 | 2021 | Singapore | Unknown | Culture | **28049** | *bla*_KPC_ | *ybt* | KL74 |
| E1011 | SRR16078953 | 2021 | Singapore | Unknown | Culture | **28235** | *bla*_NDM_ | *ybt* | KL74 |
| 2016CRE_43 | SRR13077375 | 2016 | Singapore | Human | Acute care-hospital unit | **23971** | Negative | *ybt* | KL74 |
| E183 | SRR15695711 | 2021 | Singapore | Unknown | Culture | **28121** | *bla*_OXA-48-like_ | *ybt* | KL74 |
| GASRECK144 | SRR6505387 | 2011 | Singapore | Unknown | Blood | **31613** | Negative | *ybt* | KL74 |
| ARLG-6527 | SRR14450144 | 2018 | Singapore | Human | Urine | **26762** | *bla*_OXA-48-like_ | *ybt* | KL74 |
| E650 | SRR15695360 | 2021 | Singapore | Unknown | Culture | **28011** | *bla*_OXA-48-like_ | *ybt* | KL74 |
| E504 | SRR15695853 | 2021 | Singapore | Unknown | Culture | **28161** | *bla*_OXA-48-like_ | *ybt* | KL74 |
| E123 | SRR15695965 | 2021 | Singapore | Unknown | Culture | **28191** | *bla*_OXA-48-like_ | *ybt* | KL74 |
| E219 | SRR15695397 | 2021 | Singapore | Unknown | Culture | **28016** | *bla*_OXA-48-like_ | *ybt* | KL74 |
| E60 | SRR15695444 | 2021 | Singapore | Unknown | Culture | **28033** | *bla*_OXA-48-like_ | *ybt* | KL74 |
| E1130 | SRR16078943 | 2021 | Singapore | Unknown | Culture | **28230** | *bla*_NDM_ | *ybt* | KL74 |
| E1129 | SRR16078945 | 2021 | Singapore | Unknown | Culture | **28232** | *bla*_NDM_ | *ybt* | KL74 |
| E1128 | SRR16078946 | 2021 | Singapore | Unknown | Culture | 28233 | *bla*_NDM_ | *ybt* | KL74 |
| VG117 | GCA_030128965.1 | 2020 | Portugal | Cat | **Tissue Swab** | 11775 | Negative | *ybt* | KL74 |
| CCBH27526 | GCA_016055635.1 | 2020 | Brazil | Human | Blood | 11775 | *bla*_KPC_ | *ybt* | KL74 |
| MVK-04F040 | ERR7598146 | 2019 | France | Vegetable | Salad | **5490** | Negative | Negative | KL74 |
| 51616 | SRR13356548 | 2018 | France | Bird | Culture | **10136** | Negative | Negative | KL74 |
| KPN217 | SRR5386380 | 2012 | USA | Human | Urine | **33350** | Negative | Negative | KL74 |
| 205915 | SRR17303571 | 2015 | UK | Human | **Whole organism** | **29011** | *bla*_NDM_ | Negative | KL74 |
| ATR12 | SRR9615893 | 2016 | France | Human | Urine | **32669** | Negative | Negative | KL74 |
| E437 | SRR15695949 | 2022 | Singapore | Unknown | Culture | **28184** | *bla*_NDM_ | Negative | KL74 |
| E438 | SRR16079064 | 2022 | Singapore | Unknown | Culture | 28263 | *bla*_NDM_ | Negative | KL74 |
| E1075 | SRR16079271 | 2022 | Singapore | Unknown | Culture | 28263 | *bla*_NDM_ | Negative | KL74 |
| EC0172 | SRR11992955 | 2010 | Singapore | Human | Urine | **24500** | *bla*_NDM_ | Negative | KL74 |
| M940 | DRR240452 | 2018 | Myanmar | Human | Sputum | **17083** | *bla*_NDM_ | Negative | KL74 |
| G18255128 | ERR4784384 | 2018 | India | Human | Wound | **18696** | *bla*_NDM_ | *ybt* | KL74 |
| OM49 | SRR10295028 | 2015 | Oman | Human | Wound | 14551 | *bla*_NDM_ | *ybt* | KL74 |
| RIVM_C016070 | ERR3712973 | 2020 | Netherlands | Unknown | Unknown | **20876** | *bla*_NDM_ | *iuc* | KL74 |
| NKP5 | GCA_022028955.1 | 2019 | India | Human | Blood | 14551 | Negative | Negative | KL74 |
| 71754 | SRR17302054 | 2014 | UK | Human | **Whole organism** | **28720** | *bla*_NDM_ | Negative | KL74 |
| AUSMDU00025334 | SRR15098137 | 2019 | Australia | Human | **Swab** | **11005** | Negative | Negative | KL74 |
| DHQP1301748 | SRR3996255 | 2013 | USA | Human | Urine | **31717** | Negative | *iuc* | KL74 |
| AS012481 | SRR10905613 | 2016 | USA | Human | Lung | **25417** | Negative | Negative | KL74 |
| EC1294 | SRR11622506 | 2015 | Singapore | Human | Blood | **24492** | Negative | Negative | KL74 |
| 4300STDY6636978 | ERR2397448 | 2016 | Thailand | Human | Unknown | **447** | Negative | *ybt* | KL74 |
| SCP13-52 | ERR1618832 | 2016 | Netherlands | Human | Unknown | **20744** | Negative | Negative | KL74 |
| 22110212 | GCA_030067075.1 | 2022 | China | Human | Sputum | 11162 | Negative | Negative | KL74 |
| K45-67 | SRR2598972 | 2007 | Norway | Human | Pus | **31598** | *bla*_VIM_ | Negative | KL74 |
| 110686-17 | SRR10028357 | 2017 | Switzerland | Human | Blood | **22167** | Negative | Negative | KL74 |
| 18091903568-1 | GCA_018275285.1 | 2019 | China | Human | Feces | 11162 | Negative | Negative | KL74 |
| GS888 | GCA_016879915.1 | 2016 | China | Human | Feces | 11162 | Negative | Negative | KL74 |
| 21523_3#34 | ERR1852993 | 2014 | Philippines | Human | Sputum | 19217 | *bla*_NDM_ | Negative | KL74 |
| 21523_3#37 | ERR1852996 | 2014 | Philippines | Human | Urine | 19217 | *bla*_NDM_ | Negative | KL74 |
| 21523_3#36 | ERR1852995 | 2014 | Philippines | Human | Tracheal aspirate | 19217 | *bla*_NDM_ | Negative | KL74 |
| 21523_2#114 | ERR1852887 | 2014 | Philippines | Human | Sputum | 19217 | Negative | Negative | KL74 |
| 21523_2#46 | ERR1852819 | 2013 | Philippines | Human | Sputum | 19217 | Negative | Negative | KL74 |
| 21523_2#94 | ERR1852867 | 2014 | Philippines | Human | Urine | **19350** | Negative | Negative | KL74 |
| ARPG-340 | GCA_002108235.1 | 2013 | Philippines | Human | Urine | **684** | *bla*_NDM_ | Negative | KL135 |
| ARPG-381 | GCA_002108415.1 | 2013 | Philippines | Human | CSF | **688** | *bla*_NDM_ | Negative | KL135 |
| ARPG-380 | GCA_002108405.1 | 2013 | Philippines | Human | LDT | **687** | *bla*_NDM_ | Negative | KL50 |
| ARPG-379 | GCA_002108285.1 | 2013 | Philippines | Human | CSF | **686** | *bla*_NDM_ | Negative | KL50 |
| ARPG-372 | GCA_002108245.1 | 2013 | Philippines | Human | LDT | **685** | *bla*_NDM_ | Negative | KL135 |
| G18001133 | ERR4795573 | 2017 | Colombia | Human | Urine | **19033** | *bla*_VIM_ | Negative | KL74 |
| ARLG-7407 | SRR14449682 | 2017 | Colombia | Human | Urine | **26677** | *bla*_KPC_ *bla*_VIM_ | Negative | KL74 |
| G18000677 | ERR4795649 | 2015 | Colombia | Human | Unknown | **18933** | *bla*_VIM_ | Negative | KL74 |
| AI2713 | ERR5192157 | 2016 | Spain | Human | Unknown | 18420 | *bla*_KPC_ | Negative | KL74 |
| AI2701 | ERR5216499 | 2016 | Spain | Human | Unknown | 18420 | *bla*_KPC_ | Negative | KL74 |
| G18000675 | ERR4795473 | 2015 | Colombia | Human | Blood | **18716** | *bla*_NDM_ | Negative | KL74 |
| G18000676 | ERR4795518 | 2015 | Colombia | Human | Urine | **18782** | *bla*_NDM_ | Negative | KL74 |
| COL-Kpn50 | GCA_002854655.1 | 2007 | Colombia | Human | Unknown | **1192** | Negative | Negative | KL74 |
| COL-Kpn113 | GCA_002854815.1 | 2004 | Colombia | Human | Unknown | **1147** | Negative | Negative | KL74 |
| ST273 | GCA_947390055.1 | 2017 | UK | Human | Unknown | 11161 | *bla*_NDM_ | Negative | KL74 |
| Kpn223 | SRR3465557 | 2013 | USA | Human | Unknown | **3410** | Negative | Negative | KL74 |
| MyNCGM427 | DRR198038 | 2016 | Myanmar | Human | Unknown | **19555** | *bla*_NDM_ | Negative | KL74 |
| AUSMDU00022425 | SRR15097969 | 2018 | Australia | Human | Urine | 27130 | Negative | *ybt* | KL74 |
| AUSMDU00021076 | SRR15097976 | 2018 | Australia | Human | **Swab** | 27130 | Negative | *ybt* | KL74 |
| KPN2222 | SRR18208939 | 2020 | Australia | Human | Urine | 27130 | Negative | *ybt* | KL74 |
| AUSMDU00025108 | SRR15098140 | 2019 | Australia | Human | **Swab** | **27136** | Negative | *ybt* | KL74 |
| AUSMDU00024535 | SRR15098147 | 2019 | Australia | Human | **Swab** | 27130 | Negative | *ybt* | KL74 |
| AUSMDU00030206 | SRR15098098 | 2019 | Australia | Human | **Swab** | 27130 | Negative | *ybt* | KL74 |
| FK6449 | GCA_026156565.1 | 2019 | China | Human | Blood | 27130 | Negative | *ybt* | KL74 |
| 5589 | GCA_024579815.1 | 2022 | China | Human | Blood | 11160 | *bla*_OXA-48-like_ | *ybt* | KL74 |
| E1183 | SRR15695596 | 2021 | Singapore | Unknown | Culture | 28085 | *bla*_KPC_ | Negative | KL74 |
| ENT416 | SRR9217852 | 2013 | Singapore | Human | Trachael aspirate | 28085 | *bla*_KPC_ | Negative | KL74 |
| EC0312-F-N720-1-636 | GCA_018156285.1 | 2021 | USA | Unknown | Unknown | 28085 | *bla*_KPC_ | Negative | KL74 |
| E1184 | SRR15695595 | 2021 | Singapore | Unknown | Culture | **28084** | *bla*_KPC_ | Negative | KL74 |
| 171J9 | GCA_008375015.1 | 2018 | France | Human | Culture | 28085 | *bla*_KPC_ | Negative | KL135 |
| 109-B1 | SRR8613845 | 2019 | Cambodia | Human | Feces | **31007** | Negative | Negative | KL74 |
| 2014CRE_61 | SRR13077335 | 2014 | Singapore | Human | Acute care-hospital unit | 23985 | Negative | Negative | KL74 |
| 2014CRE_18 | SRR13077449 | 2014 | Singapore | Human | Intermediate-Care Facility | 23985 | Negative | Negative | KL74 |
| 2014CRE_37 | SRR13077421 | 2014 | Singapore | Human | Acute care-hospital unit | **23957** | Negative | Negative | KL74 |
| 2014CRE_34 | SRR13077434 | 2014 | Singapore | Human | Acute care-hospital unit | **23953** | Negative | Negative | KL74 |
| 2014CRE_28 | SRR13077456 | 2014 | Singapore | Human | Acute care-hospital unit | 23985 | Negative | Negative | KL74 |
| 2015CRE_66 | SRR13077248 | 2015 | Singapore | Human | Acute care-hospital unit | **24010** | Negative | Negative | KL74 |
| E114 | SRR15695829 | 2021 | Singapore | Unknown | Culture | **28153** | *bla*_OXA-48-like_ | Negative | KL74 |
| 2014CRE_6 | SRR13077450 | 2014 | Singapore | Human | Acute care-hospital unit | 24009 | *bla*_OXA-48-like_ | Negative | KL74 |
| 2015CRE_63 | SRR13077251 | 2015 | Singapore | Human | Acute care-hospital unit | 24009 | Negative | Negative | KL74 |
| 2014CRE_58 | SRR13077369 | 2014 | Singapore | Human | Acute care-hospital unit | **23975** | Negative | Negative | KL107 |
| AUSMDU00018363 | SRR14673440 | 2018 | Australia | Human | **Fluid** | **26975** | Negative | Negative | KL74 |
| Kp_HUCA_Bac_85 | GCA_020576895.1 | 2018 | Spain | Human | Blood | 11161 | *bla*_OXA-48-like_ | Negative | KL74 |
| AUSMDU00017569 | SRR15097914 | 2018 | Australia | Human | **Swab** | **27282** | *bla*_OXA-48-like_ | Negative | KL74 |
| RBL-17-103-2 | ERR4465102 | 2020 | Germany | Human | Blood | **17581** | Negative | Negative | KL74 |
| 5012STDY7626429 | GCA_900776395.1 | 2018 | Saint Kitts | Human | Unknown | **9458** | Negative | Negative | KL74 |
| ARLG-3223 | SRR12508677 | 2016 | USA | Human | Respiratory tract | **22864** | *bla*_KPC_ | Negative | KL74 |
| ARLG-7427 | SRR14450045 | 2017 | Colombia | Human | Urine | **26793** | *bla*_KPC_ | Negative | KL74 |
| KP_NORM_BLD_116392 | ERR6293669 | 2015 | Norway | Human | Blood | **21811** | Negative | Negative | KL74 |
| KPN1526 | SRR5386648 | 2014 | USA | Human | Respiratory tract | 31728 | Negative | Negative | KL74 |
| KPN1682 | SRR5386065 | 2014 | USA | Human | Respiratory tract | 31728 | Negative | Negative | KL74 |
| KPN1544 | SRR5386633 | 2014 | USA | Human | Respiratory tract | 31728 | Negative | Negative | KL74 |
| KLPN_596 | SRR13893519 | 2018 | USA | Human | **Patient** | **25961** | Negative | Negative | KL74 |
| KLPN_574 | SRR13893522 | 2018 | USA | Human | **Patient** | **25959** | Negative | Negative | KL74 |
| KPN601 | SRR5385428 | 2013 | USA | Human | Urine | **31168** | Negative | Negative | KL74 |
| MH15-269M | GCA_011764365.1 | 2015 | Vietnam | Human | Blood | 11160 | Negative | Negative | KL74 |
| MH15-274M | GCA_011764385.1 | 2015 | Vietnam | Human | Blood | 11160 | Negative | Negative | KL74 |
| BA1688 | ERR3162567 | 2017 | India | Human | Blood | **20924** | *bla*_OXA-48-like_ | Negative | KL74 |
| 250F5 | GCA_023275135.1 | 2020 | France | Human | **Clinical sample** | 30780 | *bla*_OXA-48-like_ | Negative | KL107 |
| 6168 x2 | SRR18230960 | 2020 | Hungary | Bird | **Swab** | 30780 | *bla*_NDM_ | Negative | KL141 |
| AUSMDU00022583 | SRR14673439 | 2018 | Australia | Human | Feces | **26976** | Negative | Negative | KL10 |
| SAMEA8948947 | ERR6293693 | 2015 | Norway | Human | Blood | **21826** | Negative | *ybt* | KL177 |
| ARLG-6535 | SRR14449535 | 2018 | Singapore | Human | Urine | **26395** | *bla*_OXA-48-like_ | *ybt* | KL177 |
| E5 | GCA_026197555.1 | 2021 | China | Human | **Patient with infection** | **16326** | Negative | *ybt* | KL177 |
| HK31 | GCA_018314115.1 | 2016 | China | Human | **Whole organism** | Unknown | Negative | Negative | KL35 |
| 316 | ERR2586377 | 2012 | Vietnam | Human | Blood | **19522** | Negative | Negative | KL10 |
| TSNTC34-1 | GCA_026626065.1 | 2022 | China | Chicken | Unknown | 11160 | Negative | *ybt* | KL48 |
| WCHKP020034 | SRR6442877 | 2017 | China | Human | Culture | **2743** | *bla*_IMP_ *bla*_NDM_ | Negative | KL15 |
| 21523_2#157 | ERR1852930 | 2013 | Philippines | Human | **Arm** | **19293** | Negative | Negative | KL15 |
| MER-91 | SRR7828803 | 2016 | Singapore | Human | Blood | 27937 | Negative | Negative | KL15 |
| KP7 | SRR15342710 | 2021 | Singapore | Human | Culture | 27937 | Negative | Negative | KL15 |
| GASRECK103 | SRR6505357 | 2013 | Singapore | Human | Blood | **31504** | Negative | Negative | KL177 |
| EbB104 | ERR4019930 | 2017 | Cambodia | Human | **Clinical** | 19649 | Negative | Negative | KL15 |
| EbB105 | ERR4019931 | 2017 | Cambodia | Human | **Clinical** | 19649 | Negative | Negative | KL15 |
| 114PB | SRR10581305 | 2018 | Brazil | Cat | Nasal secretion | **25377** | Negative | Negative | KL15 |
| KI463 | DRR148483 | 2020 | Indonesia | Human | Unknown | **16952** | *bla*_NDM_ | Negative | KL15 |
| E295 | SRR16079024 | 2021 | Singapore | Unknown | Culture | **28254** | *bla*_NDM_ | Negative | KL15 |
| strain 132 | SRR4302222 | 2015 | Singapore | Human | Unknown | **32825** | *bla*_NDM_ | Negative | KL15 |
| 21523_3#148 | ERR1853107 | 2014 | Philippines | Human | Urine | 19259 | Negative | Negative | KL15 |
| 21523_3#149 | ERR1853108 | 2014 | Philippines | Human | Abscess | 19259 | Negative | Negative | KL15 |
| G18755715 | ERR4635171 | 2017 | Philippines | Human | Blood | **19209** | Negative | Negative | KL15 |
| SAMEA8948695 | ERR6294264 | 2014 | Norway | Human | Urine | 13617 | Negative | Negative | KL15 |
| A_017 | SRR15064938 | 2015 | Germany | Unknown | Throat swab | 13617 | Negative | Negative | KL15 |
| cluster8 | SRR11356417 | 2018 | Finland | Human | Unknown | **25207** | *bla*_OXA-48-like_ | Negative | KL15 |
| C046 | DRR122483 | 2015 | Thailand | Human | Blood | 13617 | *bla*_NDM_ | Negative | KL15 |
| C294 | DRR150413 | 2016 | Thailand | Human | Unknown | **16975** | *bla*_NDM_ | Negative | KL15 |
| SAMEA12549209 | ERR8098498 | 2022 | Bangladesh | Human | Blood | **24240** | *bla*_NDM_ | Negative | KL50 |
| MNH_G2C5F | GCA_030512265.1 | 2022 | Bangladesh | Cow | Feces | 29347 | Negative | Negative | KL15 |
| 128082 | SRR17308579 | 2015 | UK | Human | **Whole organism** | 29347 | *bla*_NDM_ | Negative | KL15 |

**cgST**: core-genome sequence type. **CGs**: carbapenemases genes. **VFGs**: virulence-factors genes. **CS**: capsular serotype (k-locus). **Note**: the sources of origin of the strains considered as unspecific and the cgSTs of strains not indicated in the phylogeny are indicated in bold (**Figure 5**).
